# The youth concussion awareness network (You-CAN) - a school-based peer-led intervention to improve concussion reporting and social support: the protocol for a cluster randomized trial

**DOI:** 10.1186/s12889-020-8244-5

**Published:** 2020-02-05

**Authors:** Andrea Hickling, Kylie D. Mallory, Katherine E. Wilson, Rosephine Del Fernandes, Pamela Fuselli, Nick Reed, Youth Concussion Awareness Network (You-CAN) Team, Kathryn Barnes, Stephanie Cowle, Michael J. Ellis, Ryan Hung, Anne W. Hunt, Michael G. Hutchison, Barbara Kawasoe, Gina Kay, George Kourtis, Nancy Kraetschmer, Emily Kroshus, Richard Louis, Jennifer MacPhee, Chris Markham, Christine F. Provvidenza, Kelly Russell, Shannon E. Scratch, Gideon Sheps, Brandy Tanenbaum, Paul Weiser, Roger Zemek

**Affiliations:** 1 Bloorview Research Institute, Holland Bloorview Kids Rehabilitation Hospital, Toronto, Canada; 2 Department of Occupational Science and Occupational Therapy, Faculty of Medicine, University of Toronto, Toronto, Canada; 3 Rehabilitation Sciences Institute, Faculty of Medicine, University of Toronto, Toronto, Canada; 4 Parachute, Toronto, Canada

**Keywords:** Concussion, School-based intervention, Peer education, Education, Adolescents, Brain injury

## Abstract

**Background:**

Concussion prevalence is increasing in the pediatric population, and is a matter of public health concern. Concussion symptoms can be physical, cognitive, emotional and behavioural, and last longer in high school aged youth than adults. Concussions are underreported in youth due to their lack of knowledge, social environment, perceived outcomes of reporting, norms, and self-efficacy. The Youth Concussion Awareness Network (You-CAN) is a school-based peer-led program designed to increase high school students’ intent to report a concussion, and provide social support to a peer. This study aims to investigate whether participation in You-CAN, a program grounded in service learning principles, impacts concussion knowledge, attitudes, intent to report a suspected concussion to an adult, and intent to provide social support to a peer. Secondary aims include assessing the implementation fidelity and acceptability of the intervention.

**Methods:**

This longitudinal study will use a cluster randomized trial design. Three high schools from six randomly selected Canadian school boards will participate and be randomized to three study arms: (1) You-CAN led by school staff; (2) You-CAN led by school staff and research team; and (3) untreated comparison group. Intervention arms 1 and 2 will deliver the You-CAN program and create a Concussion Council at their school. The Concussion Council will deliver a concussion awareness campaign and participate in an online showcase with other participating schools. In addition, arm 2 will have monthly video-calls with the research team. A survey based on the Theory of Planned Behaviour will be administered school-wide with all arms (1, 2, 3) at two time points (beginning {T_0_} and end {T_1_} of the school year). Exit interviews will be completed with the Concussion Councils and participating school staff.

**Discussion:**

This study will provide evidence of the effectiveness of a school-based peer-led concussion program on increasing concussion knowledge, attitudes, subjective norms, perceived behavioural control, intent to report a concussion to an adult, and intent to provide social support to a peer amongst Canadian high school students. It will also provide important information about the implementation and acceptability of the You-CAN program for high school students and staff.

**Trial registration:**

This trial is registered with the ISRCTN registry (ISRCTN64944275, 14/01/2020, retrospectively registered).

## Background

Concussion, also referred to as mild traumatic brain injury, is an injury to the brain that results from a direct blow to the head or body [1]. Concussion symptoms can be cognitive, physical, or emotional/behavioural, and can result in short and longer-term challenges [1]. Among children and youth in Canada, the prevalence of concussion has increased annually by 10.3% between 2005 and 2014 [2]. As prevalence continues to increase in the pediatric population [2, 3], concussion has been deemed a matter of public health concern [3, 4]. Concussion symptoms last longer in high school aged youth compared to younger children or adults [5]; however, high school aged youth have shown to lack basic knowledge about what a concussion is and the associated symptoms [6–8].

**Fig. 1:**
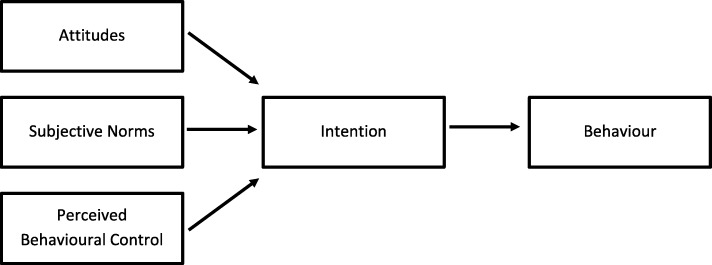
The Theory of Planned Behaviour [12]

High school aged youth often do not report a concussion because they do not understand the severity of the injury [9], do not want to let their team down [9], or do not want to appear different from their peers [10]. Increased concussion knowledge has been shown to be associated with increased concussion reporting prevalence in high school aged youth [6, 11]. The Theory of Planned Behaviour (TPB) (Fig. 1) [12] has been used to conceptualize concussion-reporting behaviour and to evaluate concussion educational initiatives [13]. The TPB states that an individual’s attitudes, subjective norms and perceived behavioural control influence their intent to perform a desired health-related behaviour, which is closely linked with actual performance of the behaviour [12]. Peers within the high school setting play an essential role in influencing individuals’ attitudes and perceived subjective norms when performing health behaviours. Youth with a history of concussion report that their peers had negative attitudes towards their concussion and lacked knowledge about how to support them [10].

School culture of support has also been identified as a key factor in promoting students’ intent to report a concussion [14]. School-based environments can be an ideal environment for promoting positive health behaviours. Peer-led education programs in school settings continue to show great promise as a tool to promote positive behaviour change [15–17]. These programs involve having peers deliver instructional or behaviour change interventions within school-based environments [15–17]. Peer educators are a similar age to the target audience and can deliver educational messages in a more relatable way than adults [15]. In addition, peer-led education programs provide opportunities for students to engage in service learning. Service learning allows students to perform community service activities while gaining valuable knowledge and leadership skills [18].

The literature calls for the implementation of concussion education programs targeted at increasing awareness, promoting reporting and creating a safe environment [6]. Caron et al. [19] identified several concussion educational programs that have been developed for youth and target their concussion knowledge; however, these interventions have almost exclusively been targeted towards athletes [20–24] or are delivered by healthcare professionals [25]. To date, no school-based, peer-led interventions have been implemented and evaluated that aim to increase the concussion knowledge, attitudes and intended behaviours of high school students.

### Goals and research hypotheses

The goal of this study is to implement and evaluate the Youth Concussion Awareness Network (You-CAN), a school-based, peer-led service learning concussion intervention for Canadian high school students to advance concussion knowledge, awareness, and access to resources. The You-CAN intervention aims to increase high school students’ intent to report a concussion to an adult and intent to provide social support to a peer following a concussion.

The study has two main hypotheses: (1) You-CAN will result in improved concussion knowledge, attitudes, subjective norms, perceived behavioural control, intent to report a concussion to an adult, and intent to provide social support to a peer amongst Canadian high school students. (2) You-CAN will be an acceptable approach to advancing concussion knowledge sharing in Canadian high schools.

## Methods/design

### Study design

**Fig. 2:**
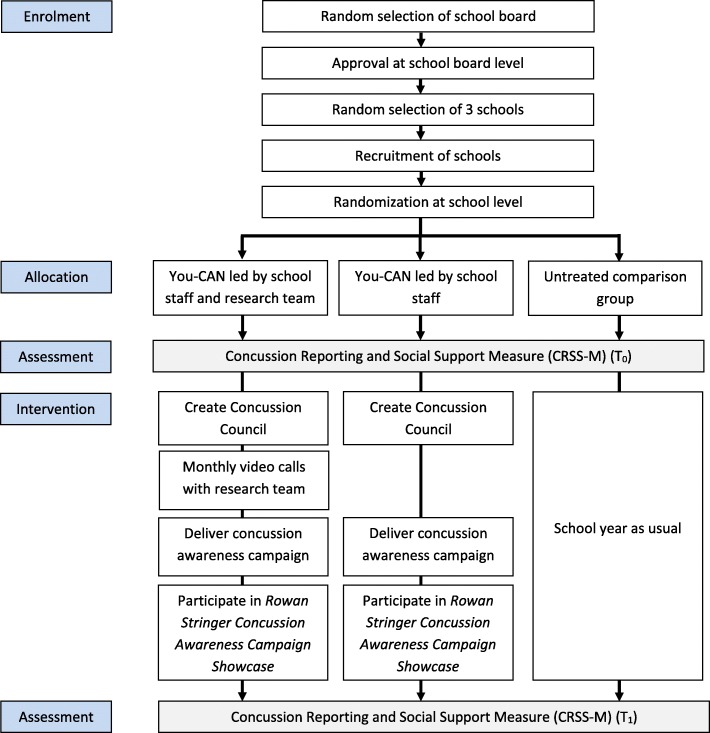
Study enrollment, allocation and intervention flow

This longitudinal study uses a cluster randomized trial design with two intervention groups and an untreated comparison group (Fig. 2). This study has been approved by the Holland Bloorview Research Ethics Board (#18–826).

### Sample size

A sample size calculation was completed based on pilot administration of this study’s main outcome measure, the Concussion Reporting and Social Support Measure (CRSS-M) (see Outcome Measures section below). The sample size was calculated using an effect size of 0.20 (α = 0.05 and power = 0.80). This is in line with previously estimated effect sizes for school-based interventions conducted with high school aged youth [26–28]. As the CRSS-M is being administered in the school-setting with support from participating school boards, principals and school staff, we expect a low attrition rate (about 10%) from the beginning {T_0_} to end {T_1_} of the school year. Based on this information, we require 140 participants to complete the survey at each participating high school.

### Participants and procedure

#### School board recruitment

Six public school boards in six Canadian provinces and/or territories will be randomly selected to participate in the study. This allows for the intervention to be evaluated with exposure to different cultures across Canada as well as both urban and rural areas. Within each province, school boards will be eligible for inclusion in the random selection process if they are primarily English-speaking and contain at least six co-ed high schools. School boards will be excluded from the random selection process if they outline an active consent process (i.e. guardian required to provide consent for student to participate) on their website. Randomly selected school boards will be invited to participate, and research applications will be submitted at the school board level. If a randomly selected school board declines participation then an additional school board will be randomly selected. This process will continue until six school boards have expressed interest and granted approval for schools within their school board to participate in the study.

#### School recruitment

After obtaining approval at the school board level, three schools within each board will be invited to participate. Schools will be included in the randomization process if they are: (1) primarily English-speaking, (2) co-ed, and (3) enrol students in grades 9–12. They will be excluded if they: (1) have less than 100 students, (2) only teach distance education, or (3) are an alternative school setting. The randomization assignment will be completed using R software [29]. All school principals from the randomly selected schools will be contacted through email and telephone via publicly available contact information in order to confirm participation and provide consent on behalf of the school. Schools will be further excluded if (1) they already have an established concussion club or (2) no other size-matched schools chose to participate.

Selected schools will be matched for school size prior to the random allocation to the study arms. School size is shown to be associated with school connectedness, students’ beliefs that teachers within the school care about them as individuals, and care about their learning [30, 31]. The following categories will be used to capture school size: (1) large population size (1000+ students), (2) medium population size (400–1000 students), and (3) low population size (less than 400 students). If three schools of matched size are not interested in participating, a different size category will be selected and invited to participate. If no size-matched schools agree to participate, then the school board will be excluded from the study and a new school board will be randomly selected to participate.

#### Intervention assignment

Once consent to participate in the study is obtained from the school principals of the three size-matched schools, a member of the research team will randomly assign schools to one of three study arms: (1) You-CAN led by school staff (school-led You-CAN); (2) You-CAN led by school staff and research team (research-led You-CAN); (3) and comparison group (See Fig. 2). The random intervention assignment will be carried out using R software [29].

Both school-led You-CAN and research-led You-CAN will implement the You-CAN intervention, with the key difference being that schools in the research-led arm will participate in monthly video calls with the research team using the video communication platform Zoom [32]. These additional video call touchpoints will result in data collected for Hypothesis 2. The school-led You-CAN arm is the more feasible version of the intervention to be implemented in schools upon study completion. The comparison group will not implement the You-CAN intervention; however, the schools will participate in the school-wide administration of the CRSS-M (see Outcome Measures section below). The comparison group will be invited to deliver You-CAN in the subsequent year, after data collection has been completed. All schools regardless of study arm will receive an honorarium for participating and a summary of the study results.

#### Student and school staff participants

Schools assigned to the school-led You-CAN and research-led You-CAN arms will deliver the intervention. Within these schools, one or two school staff members will be recruited to participate in this study. School staff will be made aware of the study through direct communication with their school’s principal. In addition, two or more students will be recruited to participate in their school’s Concussion Council, and will be made aware of the study through their school principal and participating school staff member(s). All students at the participating schools will be made aware of, and invited to participate in, the school-wide administration of the CRSS-M.

#### Informed consent

**Fig. 3:**
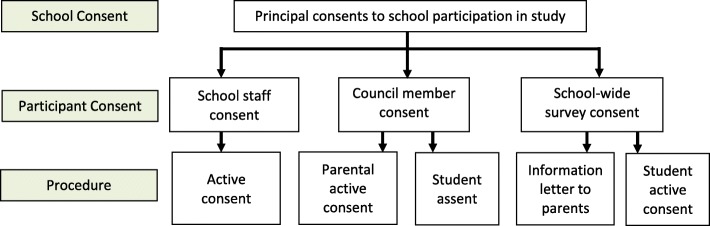
Study participant consent procedures

Figure 3 contains an overview of the study consent procedures. Principals will provide active consent on behalf of their school to participate in the study. School staff will provide active consent to participate. Parents/guardians of students participating in the Concussion Council will provide active consent for their child to participate, and students in the Concussion Council will provide assent. A school-wide information letter will be sent to all parents/guardians at the participating school to inform them of the school-wide administration of the CRSS-M, and students will actively consent to participate in the school-wide survey. Consent can be withdrawn at any time and will not affect the participant’s relationship with the school board or the research team.

### The You-CAN intervention

#### Theory

You-CAN has been developed using a service learning educational approach. According to this approach, students learn new knowledge and skills by performing community service activities [18]. Service learning is mutually beneficial to both students and the community [18, 33–35], and high school aged youth who participate in service learning have shown an increase in personal and social responsibility, transferable skills, and academic achievement [18]. Service learning allows students to learn through experience while advocating for the needs of their community [18]. The You-CAN intervention will follow the Investigation, Plan and Prepare, Action, Reflection and Demonstration (IPARD) model of service learning (Table 1) [36].

**Table 1:** The You-CAN intervention outlined using the IPARD Model of Service Learning [36]

IPARD Stage	Description
Investigate (I)	Investigate the need for concussion awareness and create concussion councils to address change
Plan and Prepare (P)	Plan and Prepare for concussion awareness campaign
Action (A)	Act by leading a week-long concussion awareness campaign
Reflect (R)	Reflect on experiences as a council member with the research team
Demonstrate (D)	Demonstrate and celebrate impact at the *Rowan Stringer Concussion Awareness Showcase*

### Delivery

#### Concussion councils

You-CAN will be delivered by a group of high school students and school staff member(s) referred to as a Concussion Council. The main goal of the Concussion Council is to plan and deliver a week-long concussion awareness campaign at their school. The Concussion Council will meet regularly (timing to be determined based on group preference) throughout the school year, and must have a minimum two students that are supported by one to two school staff. There is no maximum to the number of students who can be involved in the council.

#### You-CAN web portal

The *You-CAN Web Portal* has been developed by the research team, and contains evidence-based concussion resources [37], as well as instructions and timelines for the Concussion Council. Each participating school will have a unique login for the *You-CAN Web Portal*, allowing each Concussion Council to upload documents to their account. The *You-CAN Web Portal* will also be used as a method of communication with the research team and include functionalities such as email and video-calling. The *You-CAN Web Portal* will also contain an instructional handbook for school staff participants.

#### Concussion awareness campaign

The Concussion Council will run a week-long concussion awareness campaign. Creativity is encouraged and Concussion Councils will implement their own unique ideas that they believe will raise awareness in their school environment. There are no mandatory structured activities that must be done during the concussion awareness campaign. Any information about concussion being shared in the campaign must come from the resources on the *You-CAN Web Portal* that have been reviewed for content accuracy. If a school creates or finds a new resource for their campaign that is not on the *You-CAN Web Portal* then the resource must be approved by the research team prior to inclusion in the concussion awareness campaigns.

#### Rowan stringer concussion awareness campaign showcase

After completing a concussion awareness campaign, Concussion Councils will be invited to participate in *The Rowan Stringer Concussion Awareness Campaign Showcase.* Rowan Stringer was a high school student who passed away due to head injuries experienced playing rugby, and as a result, *Bill 193 – Rowan’s Law (Concussion Safety)* was passed in 2018 as a means to raise awareness about concussion in the province of Ontario, Canada [38]. *The Rowan Stringer Concussion Awareness Campaign Showcase* is a half-day event that will take place online through the platform Zoom [31]; with the goal of sharing and celebrating each school’s concussion awareness campaign with other participating schools from across Canada. Each school will be given 5–7 min to present highlights from their awareness campaign using a standardized PowerPoint presentation template. This portion of the intervention acts as the demonstration phase of the IPARD Model [36] outlined in Table 1.

### Outcome measures

The instruments and data collection tools selected, as well as the timeline for implementation are found in Table 2. All data will be de-identified and stored on a secure network. Only the research team will have access to the final dataset.

**Table 2:** You-CAN intervention and data collection timeline

	2019				2020				
	Sept	Oct	Nov	Dec	Jan	Feb	Mar	Apr	May
Informed consent: principals, school staff, council members	X								
Intervention time points									
Concussion awareness campaign						X	X		
*Rowan Stringer Concussion Awareness Campaign Showcase*								X	
Monthly video call with research team^*a*^		X	X	X	X	X	X	X	X
Outcome Measures									
School environment survey		X							
Concussion Reporting and Social Support Measure (CRSS-M) (T_0_)		X							
Web portal analytics		X	X	X	X	X	X	X	X
Field notes from monthly video calls^*a*^		X	X	X	X	X	X	X	X
Campaign details survey					X				
Content created					X	X	X	X	
Additional activities tracking sheet								X	
Post-showcase evaluation survey								X	
Exit interviews									X
Concussion Reporting and Social Support Measure (CRSS-M) (T_1_)									X

^*a*^*Only applies to the You-CAN led by school staff and research team arm*

### Primary outcome measures

The primary outcome of interest is the CRSS-M, which measures an individual’s intent to report a concussion to an adult and intent to provide social support to a peer who has experienced a concussion. Responses on the CRSS-M may be influenced by factors captured using the school environment survey and additional activities tracking sheet.

#### Concussion reporting and social support measure (CRSS-M)

The CRSS-M is an outcome measure developed and piloted by the research team that will be delivered to students at the beginning {T_0_} and end {T_1_} of the school year, pre- and post-participation in You-CAN. The CRSS-M was informed by the Theory of Planned Behaviour [12] and includes questions regarding demographic information (i.e. concussion history, sport participation, etc.), concussion knowledge, attitudes, perceived behavioural control, subjective norms, intent to report concussion symptoms to an adult and intent to provide social support to a peer. The CRSS-M also contains a 6 question unique identification code that allows individual surveys to be anonymously linked. It contains 57 questions including yes/no, multiple choice, checkbox, open-ended, true/false and Likert scale questions and will take approximately 10 min to complete. The CRSS-M was developed in order to provide questions about concussion that are relevant for a wide variety of high school students (sport and non-sport related questions).

#### School environment survey

The school environment survey contains questions regarding the demographics of the school population (i.e. school population, socioeconomic status, average class size) and has been developed by the research team. The school environment survey contains checkbox, yes/no, multiple choice, and open-ended response questions. This survey will be completed by school principals in all arms of the study at the beginning of the school year. This will provide important context about the different participating school environments, which will be used when interpreting CRSS-M results.

#### Additional activities tracking sheet

Concussion Councils will complete an additional activities tracking sheet that documents the dates and a brief description of any additional awareness building activities they complete outside of their week-long concussion awareness campaigns throughout the school year. This will be submitted to the research team prior to the end of the school year. Completing additional activities is not required or recommended; however, if Concussion Councils complete additional awareness building activities, it will be required for them to document the activities and will be used to provide important contextual information when interpreting CRSS-M results.

### Secondary outcome measures

The secondary outcome of the study is to understand the implementation fidelity, acceptability, and student and school staff experiences with the You-CAN intervention using the outcome measures described below.

#### You-CAN web portal analytics

Google Analytics [39] data for the *You-CAN Web Portal* domain will be downloaded on a monthly basis throughout the duration of study. This will provide data on number of users, average time on pages, location of users, operating system and devices used to access the web portal, bounce rates, behaviour flow, in-page analytics, traffic sources, form abandonment, form submissions, and how often pages were printed.

#### Field notes from monthly video calls

Field notes will be recorded using a standard template during monthly video calls with the research-led schools. Research team members will record their initial thoughts and reactions to the call, key questions asked by students, and observations. Items such as length of call, number of students present and technical issues will also be recorded.

#### Campaign details survey

The campaign details survey was developed by the research team and will be completed by Concussion Councils approximately 2 weeks prior to the delivery of their concussion awareness campaign. The survey consists of yes/no and open-ended response questions related to the planned activities for the concussion awareness campaign. Examples include: What knowledge sharing events do you have planned? Where will the event happen? Who will be involved? And what information about concussion will be provided?

#### Content created by concussion councils

As councils are encouraged to be creative when developing their concussion awareness campaigns, it is anticipated that some Concussion Councils may create new resources (i.e. videos, images, infographics) to be disseminated to their school community. These resources will be reviewed for content accuracy by the research team prior to distribution, and will be collected as data. The PowerPoint presentation that Concussion Councils create and present at the *Rowan Stringer Concussion Awareness Campaign Showcase* will also be collected.

#### Post-showcase evaluation survey

After participating in the *Rowan Stringer Concussion Awareness Campaign Showcase,* student and school staff participants will be invited to complete the post-showcase evaluation survey within 2 weeks of the event. The survey consists of thirteen Likert scale questions, one multiple choice question, and one open-ended question. The purpose of the post-showcase evaluation survey is to understand participants’ experiences and suggestions to improve the event.

#### Exit interviews

Iterative semi-structured interviews will be completed with both the Concussion Council and school staff participants. As focus group interviews have been recommended as an ideal methodology to evaluate concussion education programs [18], interviews with Concussion Council members will use a focus group format through the platform Zoom [31]. School staff interviews will be completed individually over the telephone. Interviews and focus groups will be guided by the five stages of the IPARD model [36]. The purpose of these interviews is to allow for a deeper understanding of the student and school staff experiences participating in You-CAN. Interviews will be audio recorded and transcribed verbatim.

### Data analysis

#### Hypothesis 1

To examine the first research hypothesis, the following analyses will be performed. Data from the CRSS-M will be screened for outliers, and items will be screened for multicollinearity. CRSS-M data will be included for analysis if 90% of questions per section are answered and if there are matching unique identification codes from pretest {T_0_} to posttest {T_1_}. Descriptive statistics will be used to analyze demographics, true/false, checkbox and Likert scales responses. Paired sample t-tests will be used to detect changes in individual responses at pretest {T_0_} and posttest {T_1_} time points. A repeated measure ANOVA will be used to investigate differences in the 3 study arm schools (school-led You-CAN, research-led You-CAN and comparison group) from pretest {T_0_} and posttest {T_1_}. Co-variate adjustments will be completed depending on baseline means. Change scores from each You-CAN study arm will be compared across factors using a multifactorial ANOVA. Post-hoc tests (α = 0.05) will be used to determine the mean differences between each study arm. The school environment survey data will be analyzed through a descriptive univariate analysis. To determine the influence of school factors and additional activities completed by the Concussion Councils on CRSS-M change scores, co-variate adjustments and a multifactorial linear regression will be completed. Statistical analyses will be completed using SPSS [40]. Equivalent non-parametric approaches to analyses will be used should the data not meet necessary assumptions (e.g., normal distribution etc.).

#### Hypothesis 2

To examine the second research hypothesis (H_2_), the following analyses will be performed. A descriptive univariate analysis of web portal analytic data will be completed, and compared across time. Research field notes from monthly video calls will be analyzed using categorical descriptive statistics, and content of open-ended questions will be coded and counts will be reported across coded domains. Campaign details surveys will be analyzed using descriptive statistics and frequencies. Open-ended responses will be coded and coded domains will be reported as counts (frequencies and percentages). All items created by Concussion Councils (resources, showcase presentations) will be analyzed using a content analysis [41]. This includes identifying patterns across information and visual mediums in a replicable and systematic manner, and has been used to evaluate concussion resources [42, 43]. A customized coding scheme will be developed by randomly selecting a small number of resources to be assessed prior to data analysis in order to develop and refine the coding scheme (e.g. type of image, primary content of image, purpose of image, information quality). Post-showcase survey Likert scale questions will be reported as frequencies and percentages. Open-ended questions will be coded and categorized and codes will be reported as frequencies. All exit interviews with student and school staff participants will be analyzed using a deductive thematic analysis [44]. Two independent researchers will review transcripts and generate codes across all interviews. To ensure rigour, a third member of the research team will consult at each stage of the comparative analysis to ensure the codes and themes are representative of the data. Through these analyses, information will be provided about the acceptability of the You-CAN intervention.

## Discussion

The aim of this paper is to describe the protocol for a study examining the impact and acceptability of You-CAN, a concussion education program designed to improve Canadian high school aged youths’ intent to report a concussion and provide social support to a peer with concussion. Many attempts have been made at delivering concussion education; however, most initiatives for youth are targeted solely at athletes [19–23] or are delivered by healthcare providers [25]. You-CAN is the first school-based, peer-led concussion education program and this study will make a unique contribution to literature by providing a rigorous evaluation of the program.

You-CAN has the potential to be a novel, evidence-informed school-based program for promoting concussion awareness and influencing the intended health behaviours of high school aged youth. The findings would have a significant impact for schools and school boards as they make evidence-informed decisions about how to provide concussion education to their students. If the intervention is effective at increasing concussion knowledge, attitudes, intent to report a concussion and provide support to a peer with a concussion, this could allow for the development of high school environments where youth feel socially supported and comfortable reporting potential symptoms of a concussion. Analysis of qualitative data exploring school staff and students’ experiences with the program will yield important information about the acceptability and fidelity of the current intervention. Finally, this work may also benefit other researchers who are looking to understand best practices in concussion education.

## Data Availability

Not applicable.

## Abbreviations

CRSS-M: Concussion Reporting and Social Support Measure
IPARD: Investigation, Plan, Action, Reflection, and Demonstration
TPB: Theory of Planned Behaviour
You-CAN: Youth Concussion Awareness Network
